# Supplementary material for: A Prospective Observational Study to Determine the Efficacy of a Theatre Prioritisation Tool in Optimal Utilisation of Limited Theatre Time for Deep Burn Injury in a Resource-Restricted Setting
Source: Eur Burn J. 2025 Oct 17;6(4):55. doi: 10.3390/ebj6040055 (PMC12550994; doi:10.3390/ebj6040055)
Supplement: Supplementary file 1 [file ebj-06-00055-s001.zip › Supplementary S1.pdf]

<u>Pietermaritzburg Burn Service</u>
<u>Diagnosis of Infection and Sepsis in Burns</u>

Infection and sepsis are a critical diagnosis not to miss in burn injury. The risk for development of sepsis is high due to loss of the primary barrier to infection and the immunosuppression that follows the injury. There is however no benefit attained from prophylactic antibiotics, and these should not be given. The key is early identification of sepsis which may be early (within 48 hours) and is typically toxic shock versus burn sepsis, which is typical on day 3 to 5, due to nosocomial infection. No one test is fully reliable to make the diagnosis of sepsis in the burn patient. Usually, some combination of several triggers is more helpful to initiate an action to diagnose or empirically treat sepsis in burns. The cause of the infection must be sought.
Sepsis should be considered for any the following signs:

Irritability OR Delirium OR lethargy (flat child)
Abdominal distension / Diarrhoea or vomiting = malabsorption (feed intolerance)
Temperature > 38.5 < 36 degrees
New onset / changed tachycardia or tachypnoea
Poor perfusion (delayed capillary refill, decreased GCS or decreased urine output)
Hypotension
Hypoglycaemia

In children, gastrointestinal intolerance is a sensitive sign of burn sepsis, due to ileus. This is commonly mismanaged as acute gastroenteritis and is grossly inappropriate.
In children, white cells < 6 are unlikely to be explained by anything other than sepsis and should be treated as sepsis.

There are a variety of consensus definitions for sepsis. Consideration should be given to the criteria and the complete clinical picture of the patient taken into consideration.

Consensus definitions	Criteria	Predictors
ABA Sepsis Criteria [59]	At least one or more of the following	1) Positive culture<br>2) Pathologic tissue source identified<br>3) Clinical response to antimicrobial agents
	AND at least three of the following predictors	1) Temperature >39 °C or <36.5 °C<br>2) Progressive tachycardia (>110 bpm)<br>3) Progressive tachypnoea<br>4) Thrombocytopenia<br>5) Hyperglycaemia 6) Inability to continue enteral feedings 24 hours
Mann-Salinas et al. Novel burn-specific sepsis predictors [60, 61]	Predictors	1) Tachycardia >130 bpm<br>2) MAP <60 mmHg<br>3) Base deficit <−6 mEq/l<br>4) Hypothermia <36 °C<br>5) Use of vasoactive medications<br>6) Hyperglycaemia >150 mg/dl
Sepsis-3 Consensus definition for sepsis[a] [15]	qSOFA score ≥ 2	1) Altered mental status (Glasgow Coma Scale <13)<br>2) Systolic blood pressure ≤100mmHg<br>3) Respiratory rate 22 ≥ breaths/min
	SOFA variables ≥ 2	1) PaO_2/FiO_2 ratio<br>2) Platelet count<br>3) Bilirubin<br>4) Mean arterial pressure<br>5) Glasgow Coma Scale<br>6) Vasopressor requirement<br>7) Serum creatinine or urine output
	Septic shock predictors (sepsis and both predictors)	1) Vasopressors required to maintain MAP >65mm Hg<br>2) Lactate >2 mmol/L (after adequate fluid resuscitation)

Zhang P, Zou B, Liou YC, Huang C. The pathogenesis and diagnosis of sepsis post burn injury. Burns Trauma. 2021 Feb 4;9:tkaa047. doi: 10.1093/burnst/tkaa047

Change in SOFA ≥2 points

Lactate change > 2 mmol/L (> 18 mg/dl) (a base deficit surrogate)

Temperature change – new fever or hypothermia (no consensus on threshold temperature)

Acute drop in platelet count

Urine output drop/increased fluid requirements

Kidney Disease Improving Global Outcomes (KDIGO) Acute Kidney Injury Stage ≥ 1

Respiratory changes

Alterations of mental status

Gastrointestinal dysfunction

Change in wound appearance suggestive of infection

Procalcitonin increase ≥ 2 ng/ml from initial level

Greenhalgh DG, Hill DM, Burmeister DM, Gus EI, Cleland H, Padiglione A, Holden D, Huss F, Chew MS, Kubasiak JC, Burrell A, Manzanares W, Gómez MC, Yoshimura Y, Sjöberg F, Xie WG, Egipto P, Lavrentieva A, Jain A, Miranda-Altamirano A, Raby E, Aramendi I, Sen S, Chung KK, Alvarez RJQ, Han C, Matsushima A, Elmasry M, Liu Y, Donoso CS, Bolgiani A, Johnson LS, Vana LPM, de Romero RVD, Allorto N, Abesamis G, Luna VN, Gragnani A, González CB, Basilico H, Wood F, Jeng J, Li A, Singer M, Luo G, Palmieri T, Kahn S, Joe V, Cartotto R. Surviving Sepsis After Burn Campaign. Burns. 2023 Nov;49(7):1487-1524. doi: 10.1016/j.burns.2023.05.003.
